# AKR1C3 expression in T acute lymphoblastic leukemia/lymphoma for clinical use as a biomarker

**DOI:** 10.1038/s41598-022-09697-6

**Published:** 2022-04-06

**Authors:** Deepti Reddi, Brandon W. Seaton, David Woolston, Lauri Aicher, Luke D. Monroe, Zhengwei J. Mao, Jill C. Harrell, Jerald P. Radich, Anjali Advani, Nikolaos Papadantonakis, Cecilia C. S. Yeung

**Affiliations:** 1 Department of Laboratory Medicine and Pathology, University of Washington, Seattle, USA; 2 Fred Hutchinson Cancer Research Center, Seattle, WA USA; 3 Hematology and Medical Oncology, Cleveland Clinic, Cleveland, OH USA; 4 Winship Cancer Institute of Emory University, Atlanta, GA USA

**Keywords:** Cancer, Medical research

## Abstract

To investigate aldo–keto reductase 1C3 (AKR1C3) expression in T and B acute lymphoblastic leukemia/lymphoma (ALL) patients. Three commercial antibodies were evaluated for AKR1C3 immunohistochemistry (IHC) staining performance: Polyclonal Thermofisher scientific (Clone#PA523667), rabbit monoclonal Abcam [EPR16726] (ab209899) and Sigma/Millipore anti-AKR1C3 antibody, mouse monoclonal, clone NP6.G6.A6, purified from hybridoma cell culture. Initial optimization was performed on cell line controls: HCT116 (negative control); genetically modified cell line HCT116 with AKR1C3 overexpression; Nalm and TF1 cell lines. Twenty normal bone marrows from archival B and T-ALL patient samples were subsequently examined. AKR1C3 expression levels in these samples were evaluated by immunohistochemistry, Protein Wes and quantitative RT-PCR. Sigma/Millipore Anti-AKR1C3 antibody (mouse monoclonal, clone NP6.G6.A6) showed higher specificity compared to rabbit polyclonal antibody by immunohistochemistry. H-score was used to quantify percent of nuclear immunoreactivity for AKR1C3 with varying disease involvement. T-ALL samples had a higher H-score (172–190) compared to B-ALL cases (H-score, 30–160). The AKR1C3 expression in peripheral blood by Protein Wes and RT-qPCR showed concordance in relapsed/refractory and/or minimal residual T-ALL cases. Sigma/Millipore Anti-AKR1C3 antibody and mouse monoclonal, clone NP6.G6.A6 can be used to aid in AKR1C expression of T-ALL and in cases of relapsed/refractory and/or minimal residual disease.

## Introduction

Leukemias are malignancy of hematopoietic cells based in the bone marrow and blood, and they are the most common malignancy in children, accounting for 28% of cases^1,2^. Acute lymphoblastic leukemia/lymphoma (ALL) is a malignant transformation of lymphoid progenitor cells with B and T-cell lineages ^2,3^. Although historically T acute lymphoblastic leukemia/lymphoma (T-ALL) has inferior outcomes compared to those of B acute lymphoblastic leukemia/lymphoma (B-ALL), the event-free survival has been steadily increasing with contemporary clinical trials and targeted therapy^3,4^. Despite this improvement in survival, T-ALL patients are more likely to experience induction therapy failure and early relapse^4,5^.

The gene for *aldo–keto-reductase 1C3* (*AKR1C3*) is located on the chromosome 10p15-p14, and the protein is a member of the NAD(P)H-linked oxidoreductases that reduce aldehydes and ketones to their corresponding primary and secondary alcohols^6^. By acting as a 17-ketosteroid reductase, they regulate the levels of androgens, estrogens that are available to activate receptors in peripheral tissues. AKR1C3 has been implicated in polycystic ovarian syndrome and solid organ malignancies, such as endometrial, breast and prostate cancers^6^. AKR1C3 also plays a role in non-hormonal dependent malignancies, such as acute myeloid leukemia by its role in cell proliferation and differentiation via peroxisome proliferator activated receptor γ (PPARγ) signaling pathway^7–9^. Preclinical studies have shown that T-ALL blasts show high levels of AKR1C3 and respond to PR104A and OBI-3424, both of which are small molecule prodrugs that are activated by AKR1C3^10,11^. Early clinical trials in hepatocellular carcinoma have shown efficacy when PR104 is used in combination with sorafenib in tumors with high expression of AKR1C3^12^. Polymorphism on intron 4 of the *AKR1C3* gene in maternal and offspring genotypes has been associated with increased risk of childhood leukemia^13^. This link between AKR1C3 and leukemia risk implicates a potential role of AKR1C3 in leukemogenesis^14^. We hypothesize that AKR1C3 expression can be used as a method to detect minimal residual disease in T and B-ALL patients, and patients with residual/recurrent ALL can potentially benefit from AKR1C3 targeted therapy.

## Materials and methods

### Cells lines and patient samples

Our assays were optimized and validated using four cell lines: Nalm6, HCT116, HCT116-AKR1C3, and TF1, and peripheral blood and marrow samples from patients with T-ALL (including one with ETP-ALL), B-ALL, disease-free samples from patients with histories of AML (n = 3), mantle cell lymphoma, (n = 2), and diffuse large B cell lymphoma (n = 1). Samples were obtained with patient consent from IRB approved FH biorepositories. The demographics of the patients included in our study are enumerated in Table 1. Patient samples included fresh peripheral blood, bone marrow aspirate, cryopreserved PBMC, cryopreserved BMA, and FFPE.

**Table 1:** Patient demographics.

Demographic	Result
Gender (M:F)	(33:14)^a^
Median age at biopsy	50 years old (range 20–60 years old)
**Diagnoses**	
T-ALL	25
B-ALL	24
Disease-free	6
**Marrow histology disease details**	
T-ALL disease involvement	
0–25%	10
26–50%	1
51–75%	3
76–100%	2
B-ALL disease involvement	
0–25%	2
26–50% 51–75%	7
	4
76–100%	12

Summary of patient samples from Fred Hutch biorepository.

^a^Patients whose peripheral blood and marrow samples were used for RT-qPCR or Protein Wes Simple (n = 14) were completely de-identified prior to inclusion in this study. No demographic information is available for these patients beyond their T-ALL diagnosis.

### Antibody clone selection

Three commercial antibodies were evaluated for AKR1C3 staining performance: Polyclonal Thermo Fisher Scientific (Clone#PA5-23667), rabbit monoclonal Abcam [EPR16726] (ab209899) and Sigma/Millipore Anti-AKR1C3 antibody, mouse monoclonal, clone NP6.G6.A6, purified from hybridoma cell culture. The clonal selection was a necessary first step to identify suitable reagents, technologies, detection chemistries, platforms and control tissues for assay optimization. The screening was performed on four cell line controls provided by Dr. William Wilson at University of Auckland (Supplemental Table 1): HCT116 cell line served as negative control with lack of AKR1C3; genetically modified cell line HCT116 transfected with a derivative of plasmid F527-V5; EFα promoter to overexpress AKR1C3^15,16^; Nalm6 cell line with low endogenous expression of AKR1C3; and TF1 cell line with endogenous high expression of AKR1C3.

We retrieved normal, B-ALL, and T-ALL bone marrow patient samples from Fred Hutch biorepository. AKR1C3 expression levels were validated by Western blot and IHC (protein) assay in adult patients with relapsed/refractory T-ALL, B-ALL and normal bone marrow. In addition, FFPE human thymus, tonsil, and bone marrow tissues, spleen, small intestine, colon, tongue, liver, pancreas, adrenal, testis, ovary, prostate, brain, hypophysis, kidney, and breast were procured in the Departments of Pathology, Seattle Cancer Care Alliance Center. Human tissue specimens were obtained with approval by the Fred Hutchinson Cancer Research Center Institutional Review Board (IRB) and with informed consent from all participants, with the study being conducted in accordance with IRB guidelines and regulations and in accordance with the Declaration of Helsinki.

### Immunohistochemistry

Tissue sections were cut 4 μm in thickness on positively charged slides (Fisher superfrost plus). Slides were then baked, deparaffinized, and antigen retrieval was performed with 1 × Tris–EDTA retrieval buffer for 15 min at 110 °C. Immunostains were performed on Biocare IntelliPath IHC automated slide stainer. Concentrated AKR1C3 antibody was diluted to 2 µg/mL into Biocare Devinci diluent, and a minimum of 300 µL is required per slide. Quality Control tissue was included in every run of AKR1C3 and contained elements known to be both positive and negative for AKR1C3 staining. AKR1C3 protein positivity was defined as nuclear and or cytoplasmic staining of cells. AKR1C3 positivity was graded as dim (1 +), moderate (2 +) or strong (3 +) in expression levels with a H-score. Normal lymphocytes are negative and AKR1C3 immunohistochemistry (IHC) is interpreted as negative when staining was below the expression level of normal background lymphocytes.

### Cell lysates, RNA extractions

Cells were isolated after red cell lysis and treated with quick snap freeze method to generate cell lysates. White blood cells (WBCs) were separated from 1 to 3 mL of bone marrow aspirate (BMA) or 3–5 mL of peripheral blood (PB), drawn in EDTA-containing blood collection tubes, using the WBC Separation by RNA Lysis procedure, and placed into TRIzol. RNA was extracted from the separated WBCs following the TRIzol RNA Extraction protocol, and RNA concentrations were normalized to 100 ng/µL.

### Western blot method

We tested archival T-ALL samples from Fred Hutch repository and clinical flow cytometry samples of prospective T-ALL patient samples were used to calculate an appropriate threshold for Protein Wes simple in our analysis. We compared AKR1C3 expression by Protein Wes to RNA expression by RT-PCR. Protein concentration was measured using the BioRad Protein Assay, and input volumes were normalized respectively. AKR1C3 was measured on the Protein Wes sample with the mouse monoclonal NP6.G6.A6 antibody (Sigma/Millipore) according to manufacturer’s protocol.

### Quantitative reverse transcription PCR

RT-qPCRs were performed following the protocol for the TaqMan® RNA-to-CT™1-Step Kit and run on the BioRad CFX 384 Real Time PCR Detection System. The assay’s forward primer sequences were GAGAAGTAAAGCTTTGGAGGTCACA, and the reverse sequences were CAACCTGCTCCTCATTATTGTATAAATGA and CACCCATCGTTTGTCTCGT. Samples derived from cell lines A549 and HCT116 were used as high and low positive controls for AKR1C3 expression, and Special Reagent Water was used as both the RNA-negative extraction control and the no-template control (NTC).

## Results

### AKR1C3 expression in cell lines by IHC compared with protein wes

**Figure 1:**
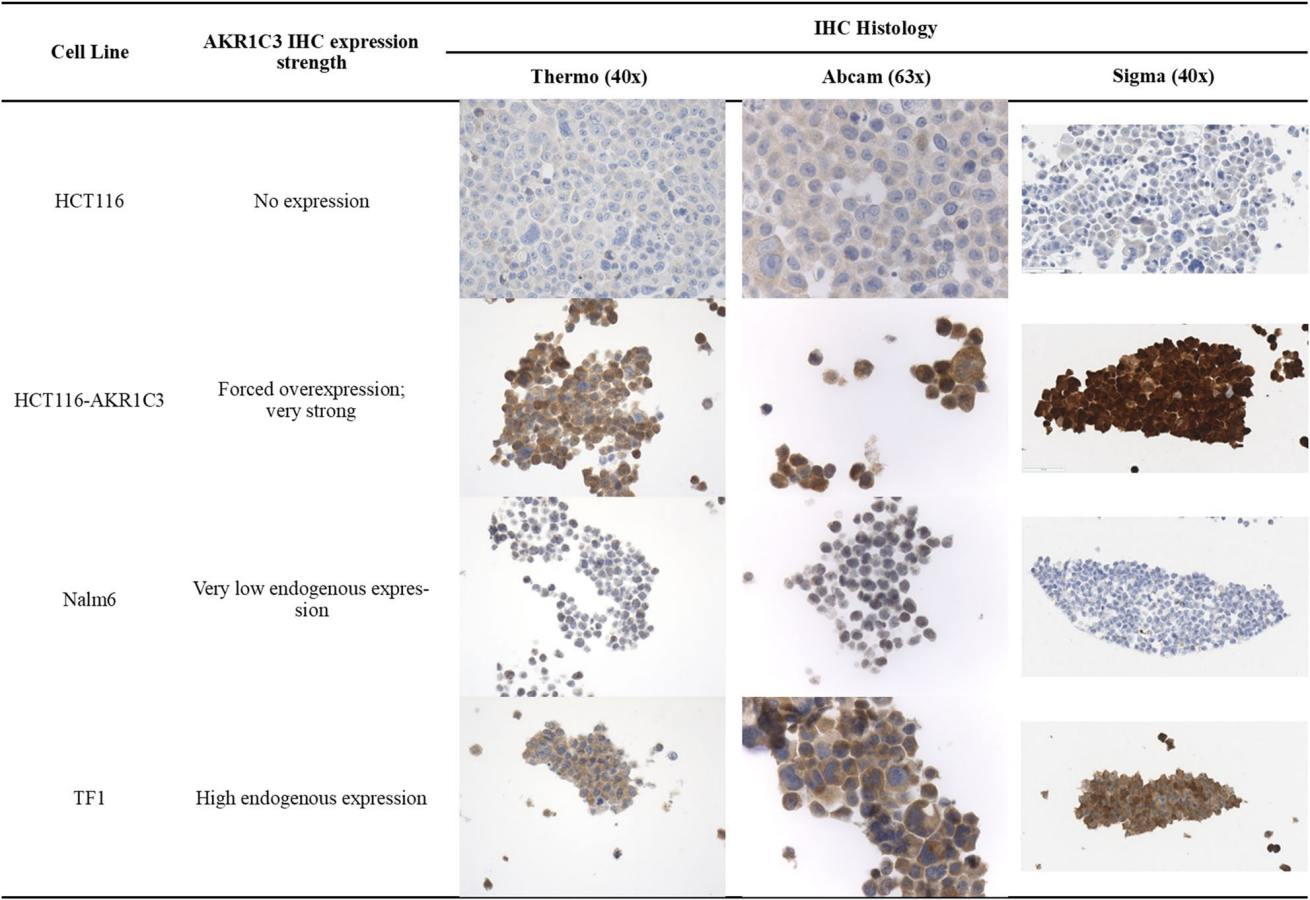
AKR1C3 immunoreactivity of four antibodies on control cell lines. Immunoreactivity of Thermo Fisher Scientific (Clone#PA5-23667), rabbit monoclonal Abcam [EPR16726] (ab209899) and Sigma/Millipore Anti-AKR1C3 antibody, mouse monoclonal, clone NP6.G6.A6, on different control cell lines. HCT116 is a human colorectal cancer cell line. HCT116-AKR1C3 is HCT116 engineered overexpress AKR1C3. Nalm6 is a Pre-B cell ALL cell line with very low endogenous AKR1C3 expression. TF1 is an erythroleukemia cell line with high endogenous AKR1C3 expression.

By screening cell-line controls, Anti-AKR1C3 mouse monoclonal NP6.G6.A6 antibody was chosen over rabbit polyclonal PA5-23667 antibody for the study based on nonspecific reactivity of PA5-23667 (Supplemental Table 2). In the second set of experiments, serial titration of NP6.G6.A6 antibody at 0.1, 0.2 and 0.4 µg/mL and of protein extract concentrations at 5, 20 and 100 µg/mL from A549 and HepG2 cell lines yielded optimal concentrations of 0.2 µg/mL and 20 µg/mL, respectively for detection via Protein Wes (Supplemental Fig. 1). In a final series of experiments on AKR1C3 expression in a series of cell lines with published results, we showed that the NP6.G6.A6 antibody can capitulate expected expressions (Fig. 1)^16,17^.

### AKR1C3 immunohistochemistry development and performance characteristics in T and B-ALL

**Figure 2:**
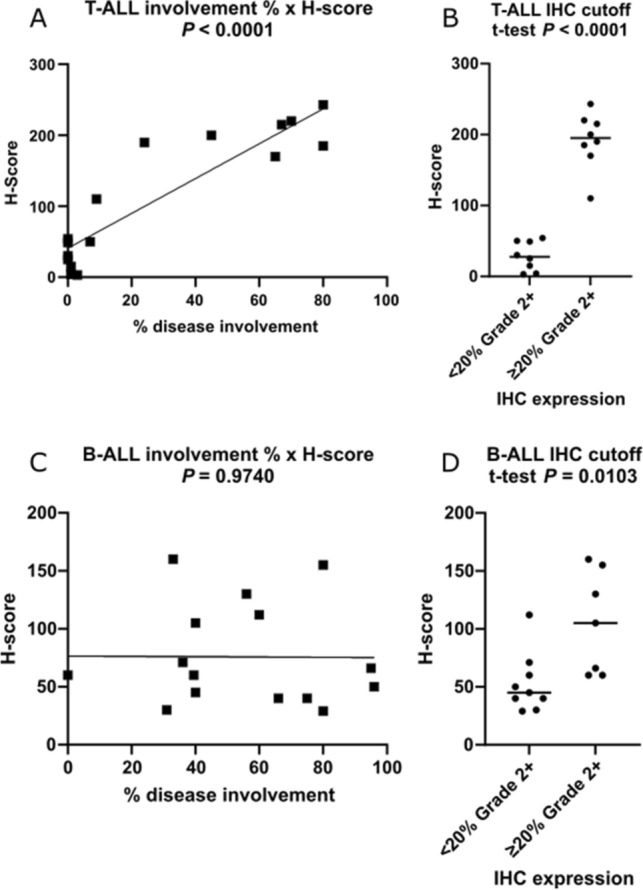
AKR1C3 IHC H-score correlation with ALL blast percentage. Quantification of singleplex nuclear immunoreactivity of AKR1C3 by the H-score in B and T lymphoblastic leukemia/lymphoma among cases with IHC expression of < or ≥ 20% Grade 2 +. Correlation with disease involvement is significant in T-ALL, but not B-ALL.

AKR1C3 IHC was optimized on cell lines with known expression levels that were confirmed in our Protein Wes experiments. AKR1C3 IHC assay was then applied to archived clinical T-ALL marrow specimens to demonstrate a positive correlation of disease involvement with specimens’ corresponding H-scores (*P* < 0.0001) (Fig. 2A,B). This contrasts with B-ALL disease involvement which correlates very poorly with corresponding H- scores (*P* = 0.9740) (Fig. 2C), although specimens could be segregated by the ≥ 20% 2 + cutoff with statistical significance (*P* = 0.0103) (Fig. 2D). The 2 × 2 analysis of the ≥ 20% 2 + cutoff in T-ALL, using the WHO definition of ≥ 20% blast involvement, yielded 100% sensitivity of 88.9% specificity for singleplex AKR1C3 IHC assay in T-ALL (Table 2). Thus, we established a cutoff of ≥ 20% blasts with at least medium strength (≥ 20%, 2 +) as our threshold for AKR1C3 positivity. Parallel analysis for B-ALL yielded 28.57% sensitivity (if IHC cutoff is ≥ 20% 2 +) or 35.71% sensitivity (if IHC cutoff is ≥ 20% 2 +). B-ALL specificity could not be calculated because our cohort contained no marrows with < 20% blast involvement.

**Table 2:** AKR1C3 Singleplex IHC 2 × 2 analysis in T-ALL.

IHC cutoff ≥ 20% gr2	≥ 20% ALL blast %		
	Positive	Negative	Total
Positive	7	1	8
Negative	0	8	8
Total	7	9	16

Compared to the percentage of T-ALL disease involvement in the marrow AKR1C3 IHC sensitivity = 100%, and specificity = 88.9%.

RT-qPCR testing of FFPE specimens run in parallel with singleplex IHC neither correlated with morphologic tumor burden (Supplemental Fig. 2A) nor H-scores (Supplemental Fig. 2B), nor was there any statistical distinction in RT-qPCR expression between samples with < or ≥ 20% 2 + IHC staining (Supplemental Fig. 3).

### AKR1C3 expression by RT-qPCR and protein wes in T-ALL peripheral blood and marrow aspirate specimens

**Figure 3:**
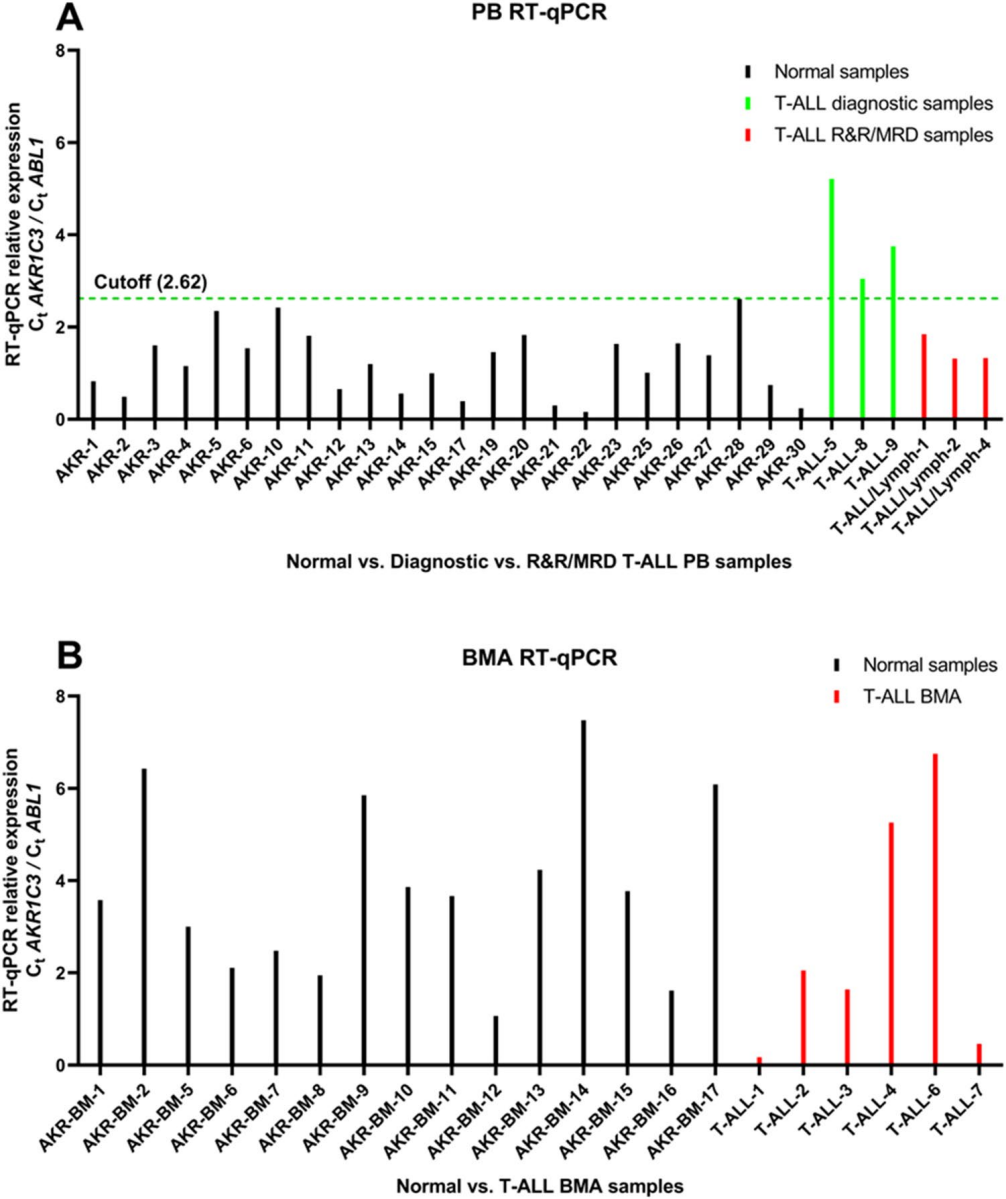
AKR1C3 RT-qPCR in blood and bone marrow. RT-qPCR values of diagnostic peripheral blood (**A**, green), R&R/MRD peripheral blood (**A**, red), and marrow aspirates (**B**, red). Diagnostic peripheral blood samples surpassed the calculated cutoff value (Table 3).

AKR1C3 RNA Expression assay yielded a reference range of 0.16–2.61 with a 2 × standard deviation of 1.4 (Table 3). Three diagnostic T-ALL specimens and three MRD/post-treatment T-ALL specimens showed expected AKR1C3 levels (Table 3), with all diagnostic specimens expressing AKR1C3 significantly more than the MRD/post-treatment T-ALL specimens (Fig. 3). Based on this analysis, an AKR1C3 RNA expression threshold of 2.62 (normalized ratio to *ABL1*) to differentiate diagnostic T-ALL specimens and this MRD/post-treatment would give us a t-test *P* value of 0.0001, with a 95% confidence interval from − 3.733065 to − 1.854085. Based on these results, we do not recommend use of this assay for testing in T-ALL bone marrow samples and where ALL disease burdens are < 20%.

**Table 3:** AKR1C3 RT-qPCR cutoff values in peripheral blood and bone marrow samples.

Category	Mean	Range	SD	2SD	Cutoff
Peripheral blood molecular mean	2.75	1.31–5.21	1.56	3.10	n/a
Diagnostic T-ALL peripheral blood molecular mean	4.0013	3.05–5.21	n/a	n/a	n/a
MRD/low level residual T-ALL peripheral blood molecular mean	1.3218	1.21–1.85	n/a	n/a	n/a
Peripheral blood molecular cutoff (normal controls)	1.21	0.16–2.61	0.71	1.41	2.62 (*P* < 0.0001)
Bone marrow molecular mean	2.72	0.17–6.74	2.68	5.36	n/a
Bone marrow molecular cutoff (normal controls)	3.81	1.06–7.47	1.91	3.82	7.61 (*P* = 0.31)

Means, ranges, standard deviations, and cutoffs of the data illustrated in Fig. 3. A statistically significant relative expression cutoff relative expression of 2.62 is observed among peripheral blood specimens, but not for bone marrow samples. Cutoff values were calculated against negative controls.

**Figure 4:**
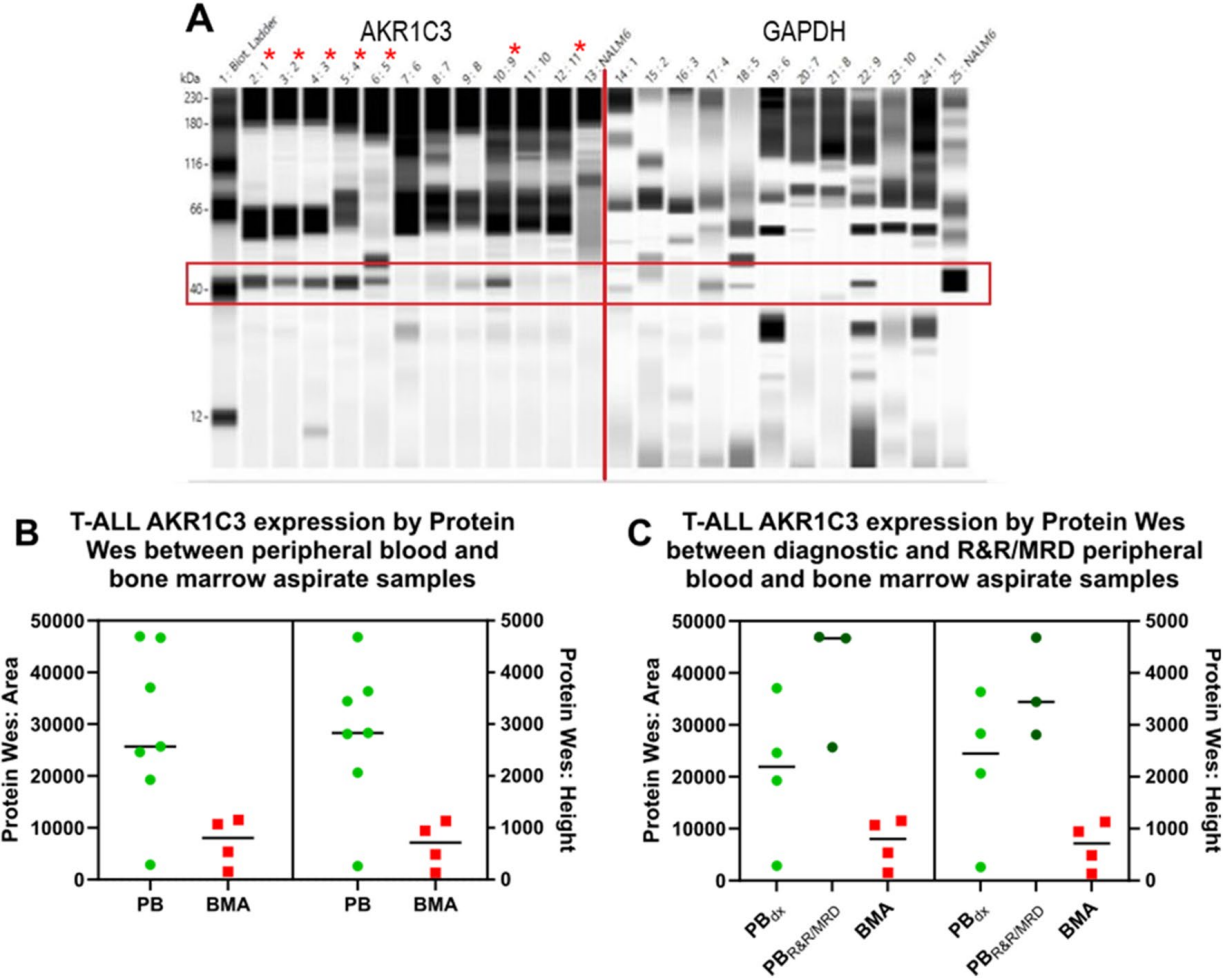
AKR1C3 expression by Protein Wes Simple in blood and bone marrow. (**A**) Increased expression of AKR1C3 by Protein Wes in peripheral blood specimens (indicated by red *) compared to the marrow aspirate specimens and control GAPDH expression. (**B**) In T-ALL cases with AKR1C3 expression in peripheral blood (PB) versus bone marrow aspirate (BMA), in which elevated expression is observed in PB samples regardless of their timepoint (Protein Wes Area t-test: P = 0.0277; Protein Wes height t-test: P = 0.0166). (**C**) When PB samples were segregated into diagnostic (PBdx) and samples of relapsed/refractory and/or minimal residual disease (PBR&R/MRD), PBR&R/MRD samples demonstrated elevated expression compared to BMA (Protein Wes Area ordinary one-way ANOVA: P = 0.0003).

11 T-ALL samples (5 whole or unsorted PB specimens and 6 flow-sorted) were Protein Wes tested and the result was compared with RNA expression by RT-qPCR. This was used to calculate an appropriate threshold for Protein Wes (Fig. 4A). Based on the data (Supplemental Table 3) we proposed setting the threshold at 10,000 which would provide a sensitivity of 70%. Specificity was incalculable from this data. The three false negative samples that would result from this threshold are Patient 6, which is a flow sorted sample with very low cell counts (1.5 × 10^6^ cells); Patient 10, another flow sorted sample with very low cell counts (8 × 10^6^ cells); and Patient 11, also a flow sorted sample with very low cell counts (5.34 × 10^6^ cells). All three samples were flow sorted 48–72 h after collection, frozen and then tested months later, therefore we do not recommend this to be performed as part of the routine protocol.

Further comparison of AKR1C3 expression by Protein Wes between PB specimens and BMA specimens demonstrated significantly greater expression in PB specimens than BMA specimens (Fig. 4B). However, no statistically significant distinction could be made between diagnostic and MRD/post-treatment specimens by Protein Wes as was observed by RT-qPCR (Fig. 4C). Elevated AKR1C3 expression was observed in PB samples regardless of the time point observed.

## Discussion

We demonstrated an orthogonal approach to validating expression assays for AKR1C3. In our Protein Wes studies, we have shown superior performance of antibody clone NP6.G6.A6, on cell lines we have demonstrated high specificity of our Protein Wes and antibody. On patient samples we showed comparative performance of IHC results were as high-performance characteristics in T-ALL patients and would be useful in identifying those who may benefit from AKR1C3 dependent therapies in contrast to B-ALL. However, we also demonstrated that RT-qPCR would be a good alternative approach for AKR1C3 expression analysis if a liquid biopsy from peripheral blood were the only available specimen in T-ALL patients with ≥ 20% circulating blasts. Peripheral blood was identified as the ideal tissue over bone marrow in such circumstances, likely because of normal marrow cells expressing AKR1C3 (e.g., erythroid progenitor cells, which generally do not circulate in peripheral blood) confounding the molecular assay results and reducing assay sensitivity. In patients where bone marrow biopsy is not feasible, evaluation of fine needle aspirations/biopsies from other tissue sites (i.e., pleural fluids or lymph nodes) with expression of AKR1C3 would be helpful in establishing the diagnosis of T-ALL.

Characterization of AKR1C3 in acute leukemia is important because it can serve as a marker for potential therapeutic and detection of minimal residual disease. Moradi Manesh et al., reported that T-ALL xenografts showed higher levels of AKR1C3 expression, which in turn made the cells more sensitive to a prodrug of nitrogen mustard, which is shown to have efficacy in patients with relapsed/refractory disease^10^. In our study we have validated the use of mouse monoclonal antibody, NP6.G6.A6, in human tissues and the AKR1C3 protein expression level by IHC correlated with Protein Wes. When comparing the IHC H-score and RT-qPCR of AKR1C3 expression to the disease involvement of the marrow, there is a positive correlation with T-ALL compared to B-ALL. During evaluation of marrow biopsy samples, we have noted that there is cross reactivity of the antibody with cells from the erythroid lineage, which can be used as a potential therapeutic target for myeloproliferative neoplasms.

There are potent and highly selective AKR1C3 inhibitors for the treatment of acute myeloid leukemia and T-ALL^11,18^. In cell lines they have been shown to have synergetic effect in combination with therapeutics daunorubicin and cytarabine^18^. In our study it is interesting that the AKR1C3 expression differs in peripheral blood and bone marrow specimens. There is a consistently high level of AKR1C3 expression in peripheral blood in patients during initial diagnosis, refractory/ residual and MRD specimens. Further study is necessary to evaluate if the AKR1C3 inhibitors are selectively targeting blasts in peripheral blood and are not affective in aleukemic T-ALL patients.

There are reports that showed AKR1C3 enzymes correlate with response to T-ALL therapy^19^. In vitro and in vivo xenograft studies have shown that AKR1C13 enzymes are overexpressed in patients with persistent disease despite therapy. In both our Protein Wes and RT-qPCR samples we have shown persistent elevation in AKR1C3 expression in patients with refractory/recurrent disease and MRD. There is potential use of AKR1C3 as a molecular assay for the evaluation of minimal residual disease during bone marrow transplantation. AKR1C3 testing is currently recommended as a method of determining patients’ potential benefit from a specific class of small molecule drugs activated by the enzyme Aldo–keto reductase 1C3, and not to be used for MRD monitoring. As our studies required ≥ 20% blasts for the evaluation of AKR1C3 by protein expression, we do not recommend use of this assay for testing in T-ALL samples with only MRD or low levels of residual disease. More sensitive assays such as flow cytometry or in certain cases sequencing of immunoglobulin heavy chain genes are recommended for MRD monitoring.

A limitation highlighted by this study was the expression of AKR1C3 in some monocytes and erythroid progenitors which could be a confounding factor. More study is needed as to how AKR1C3 expression affects therapy with the AKR1C3 activated small molecules and if off targeted toxicities could be a possibility. We have also demonstrated that in the case of a single plex IHC assay or RT-qPCR assay with marrow our sensitivity would be a limiting factor for specimens with a minimum disease burden of < 20%, future studies underway include multiplexed immunohistochemistry with improved sensitivity.

In conclusion, AKR1C3 is a potential useful biomarker as AKR1C3 is both a marker of disease burden and a therapeutic target. We have demonstrated two validated assays, both RT-qPCR and Protein Wes, which showed increased AKR1C3 expression in both PB and BMA of T-ALL specimens. Future studies with a larger patient cohort will further validate the use of AKR1C3 in clinical settings. In addition, more studies in AKR1C3 targeted therapy will help improve cancer care in patients with T-ALL.

## Supplementary Information

Supplementary Figure 1.

Supplementary Figure 2.

Supplementary Figure 3.

Supplementary Table 1.

Supplementary Table 2.

Supplementary Table 3.
