# Supplementary figures and images for: AKR1C3 expression in T acute lymphoblastic leukemia/lymphoma for clinical use as a biomarker
Source: Sci Rep. 2022 Apr 6;12:5809. doi: 10.1038/s41598-022-09697-6 (PMC8986791; doi:10.1038/s41598-022-09697-6)

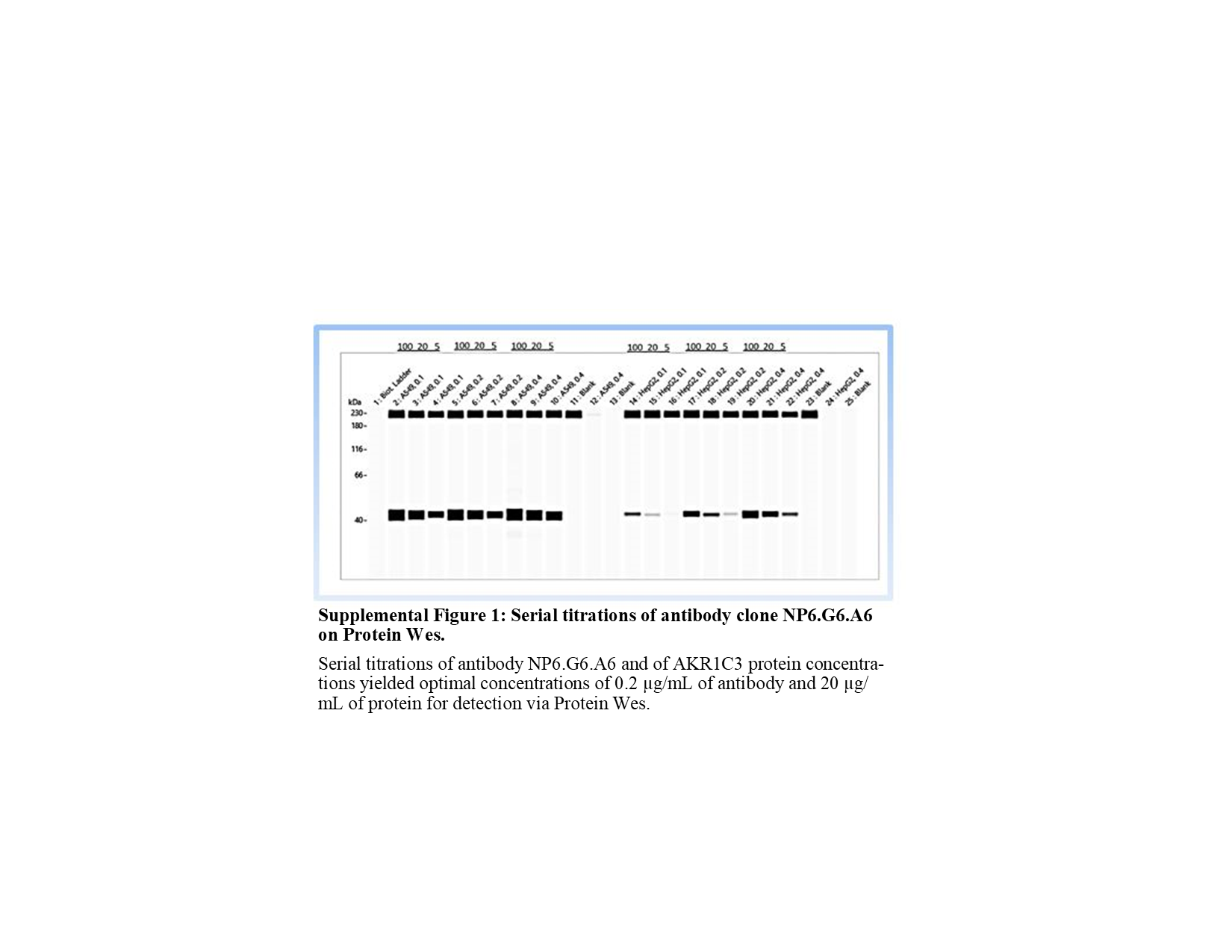

Supplement: Supplementary file 1 — Supplementary Figure 1. [file 41598_2022_9697_MOESM1_ESM.png]

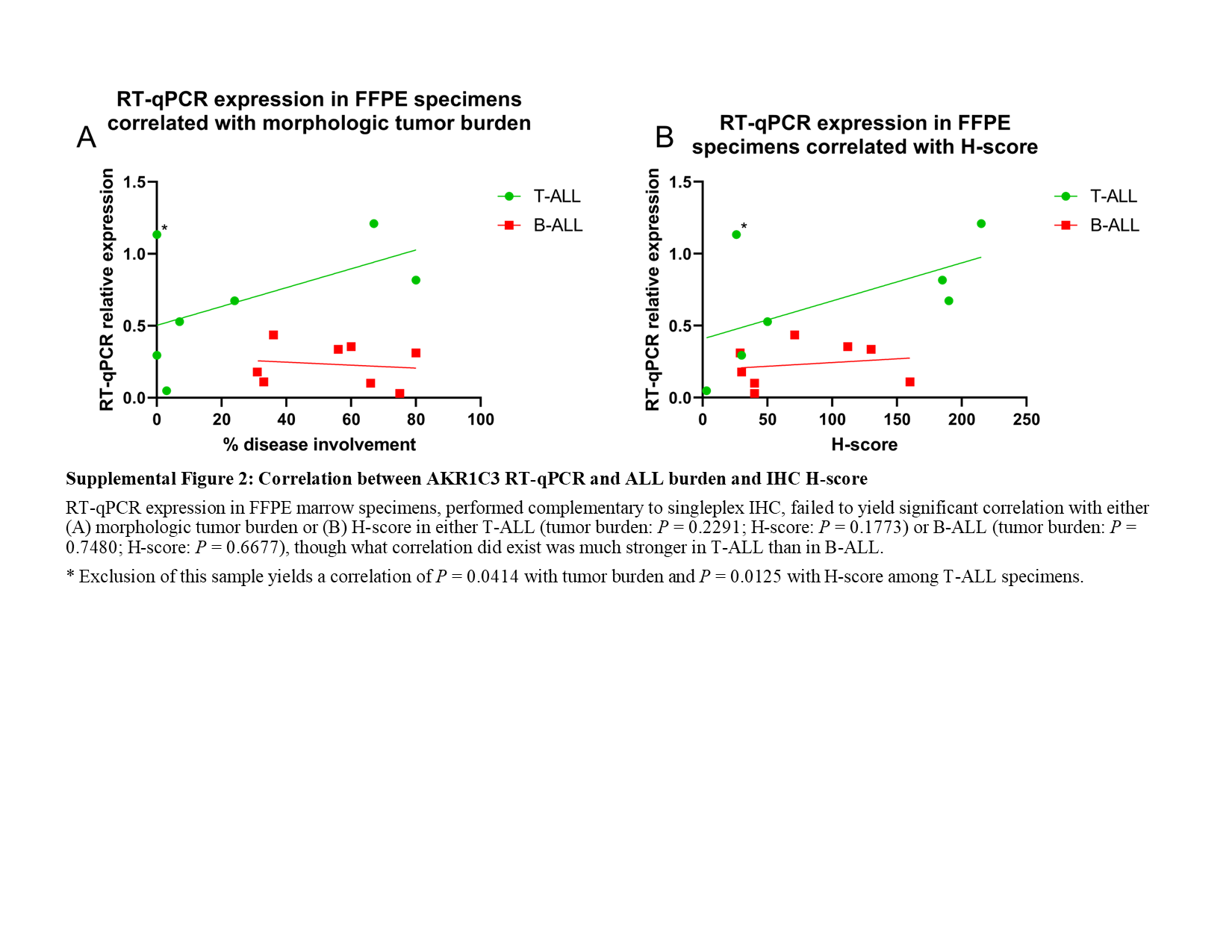

Supplement: Supplementary file 2 — Supplementary Figure 2. [file 41598_2022_9697_MOESM2_ESM.png]

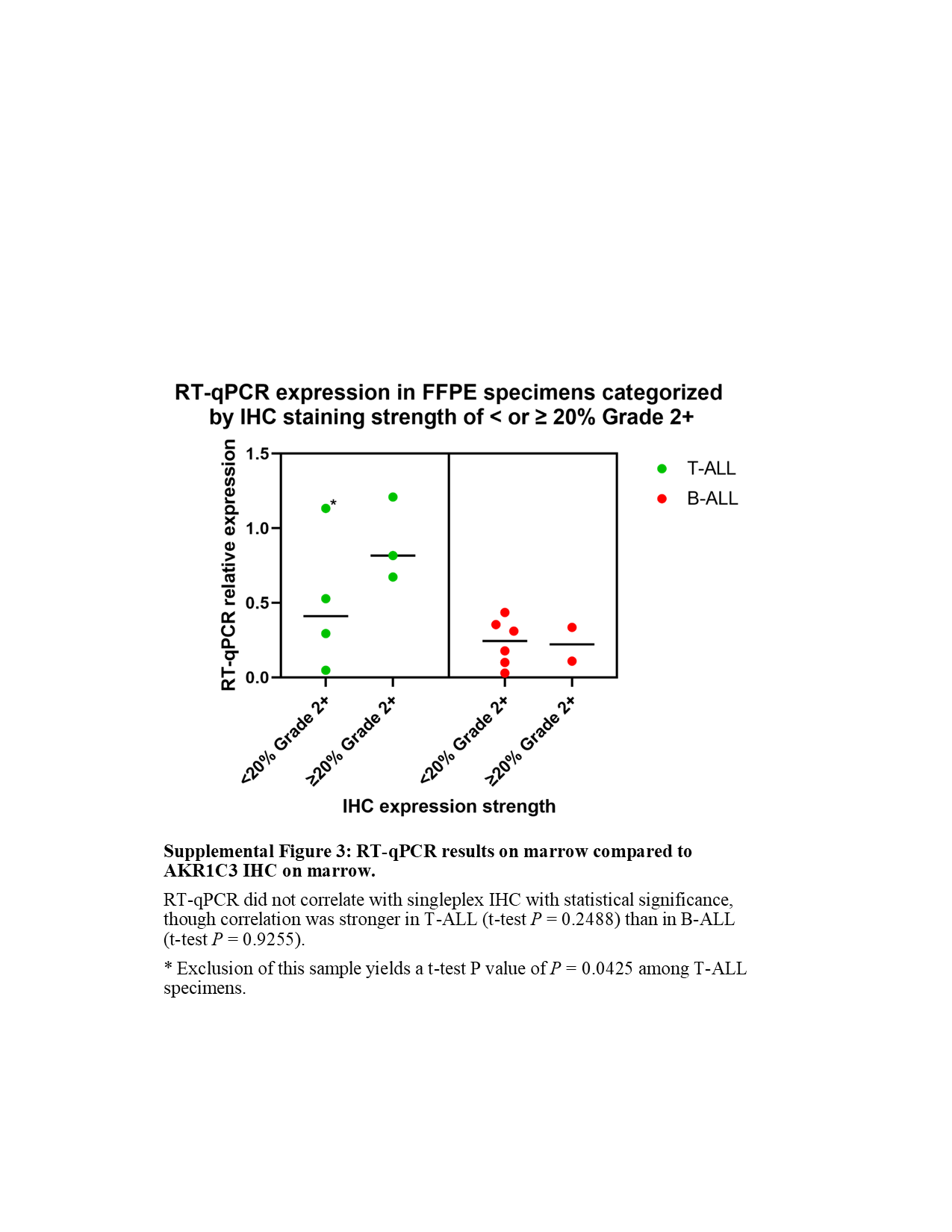

Supplement: Supplementary file 3 — Supplementary Figure 3. [file 41598_2022_9697_MOESM3_ESM.png]
