# Supplementary material for: AKR1C3 expression in T acute lymphoblastic leukemia/lymphoma for clinical use as a biomarker
Source: Sci Rep. 2022 Apr 6;12:5809. doi: 10.1038/s41598-022-09697-6 (PMC8986791; doi:10.1038/s41598-022-09697-6)
Supplement: Supplementary file 4 — Supplementary Table 1. [file 41598_2022_9697_MOESM4_ESM.docx]

**Supplemental Table 1:** **Control cell lines utilized for antibody screening and assay optimization.** Provided by Dr. William Wilson of the University of Auckland.

| **Cell line name** | **Description** | **Original cell line source** | **Morphologic description** |
| --- | --- | --- | --- |
| **Nalm6** | Pre-B cell ALL (very low endogenous AKR1C3) | Dr Marina Konopleva, MDACC | Small-medium sized cells in cohesive sheets with scant cytoplasm |
| **TF1** | Erythroleukemia (high endogenous AKR1C3) | Dr Julian Down, MIT | Medium-large sized cells in cohesive sheets with moderate cytoplasm |
| **HCT116** | Parental colon cancer line | ATCC | Pleomorphic cells with prominent nucleoli in cohesive sheets with ample cytoplasm |
| **HCT116/**  **AKR1C3** | HCT116 overexpressing AKR1C3. (Transfected with a derivative of plasmid F527-V5; EF1α promoter) | Guise et al., Cancer Res 70: 1573-84, 2010. Also used in Jamieson et al., *Biochem Pharmacol* 88: 36-45, 2014 | Pleomorphic cells with prominent nucleoli in cohesive sheets with ample cytoplasm |
