# Supplementary material for: AKR1C3 expression in T acute lymphoblastic leukemia/lymphoma for clinical use as a biomarker
Source: Sci Rep. 2022 Apr 6;12:5809. doi: 10.1038/s41598-022-09697-6 (PMC8986791; doi:10.1038/s41598-022-09697-6)
Supplement: Supplementary file 5 — Supplementary Table 2. [file 41598_2022_9697_MOESM5_ESM.docx]

**Supplemental Table 2: Immunoactivity of AKR1C3 antibodies in various cells lines on Protein Wes Simple.** Mouse monoclonal antibody NP6.G6.A6 by Sigma / Millipore demonstrates the expected immunoreactivity expression pattern of all cell lines tested, whereas than rabbit polyclonal antibody PA5-23667 by Thermo Fisher Scientific demonstrated nonspecific immunoreactivity in all cell lines.

| **cc antibody** | **Cell line** | | | | | | |  |
| --- | --- | --- | --- | --- | --- | --- | --- | --- |
|  | **K562** | **HCT116** | **MOLT4** | **A431** | **Fugi** | **Hu fibroblast** | **Hu Liver** |  |
| NP6.G6.A6 (Ms mAB) | ++ | ++ | ++ | ++ | -/+ | ++++ | ++++ |  |
|  |  |  |  |  |  |  |  |  |
| PA5-23667 (Rb poly) | not specific | not specific | not specific | not specific | not specific | not specific | not specific |  |
|  |  |  |  |  |  |  |  |  |
