# Supplementary material for: AKR1C3 expression in T acute lymphoblastic leukemia/lymphoma for clinical use as a biomarker
Source: Sci Rep. 2022 Apr 6;12:5809. doi: 10.1038/s41598-022-09697-6 (PMC8986791; doi:10.1038/s41598-022-09697-6)
Supplement: Supplementary file 6 — Supplementary Table 3. [file 41598_2022_9697_MOESM6_ESM.docx]

**Supplemental Table 3. Tabulated Protein WES and RT-qPCR results.** Protein Wes and Molecular result values in diagnostic peripheral blood, R&R/MRD peripheral blood, and marrow aspirate.

| **SDS PAGE Well #** | **Sample type** | **Cells/vial (10^6)** | **% Abnormal mononuclear cells** | **Protein Wes data** | | | **Molecular Result** |
| --- | --- | --- | --- | --- | --- | --- | --- |
|  |  |  |  | **Molecular Weight (kDa)** | **Protein Wes: Height** | **Protein Wes: Area** |  |
| 1 | PB* | n/a | n/a | 43 | 3636.4 | 37067.9 | 3.749 |
| 2 | PB* | n/a | n/a | 43 | 2065.7 | 19263.8 | 0.111 |
| 3 | PB | n/a | n/a | 42 | 2808.8 | 25697.9 | 1.846 |
| 4 | PB | n/a | n/a | 42 | 4680.3 | 46686.5 | 1.317 |
| 5 | PB | n/a | n/a | 49 | 3443.1 | 46940.5 | 0.175 |
| 6 | BM | 1.5 | 0.978 | 44.2 | 129.6 | 1500 | 0.172 |
| 7 | BM | 8 | 0.985 | 42 | 941.7 | 10692 | 1.642 |
| 8 | BM | 9.5 | 0.9 | 41.4 | 1130.5 | 11503.6 | 5.258 |
| 9 | PB | 10 | 0.981 | 42 | 2861.2 | 24588.4 | 5.210 |
| 10 | BM | 8 | 0.681 | 41.1 | 486.9 | 5371.9 | 6.749 |
| 11 | PB** | 5.34 | 0.84 | 42.1 | 262.1 | 2838.1 | 3.046 |
| n/a | BM | n/a | n/a | n/a | n/a | n/a | 2.051 |
| n/a | BM | n/a | n/a | n/a | n/a | n/a | 0.458 |
| n/a | PB | n/a | n/a | n/a | n/a | n/a | 1.327 |

* Fresh frozen

** Sorted
